# Cryoprotective Potential of Theobromine in the Improvement of the Post-Thaw Quality of Bovine Spermatozoa

**DOI:** 10.3390/cells13201710

**Published:** 2024-10-16

**Authors:** Filip Benko, Štefan Baňas, Michal Ďuračka, Miroslava Kačániová, Eva Tvrdá

**Affiliations:** 1 Institute of Biotechnology, Faculty of Biotechnology and Food Sciences, Slovak University of Agriculture in Nitra, Tr. A. Hlinku 2, 949 76 Nitra, Slovakia; filip.benko@uniag.sk (F.B.); stfnbanas@gmail.com (Š.B.); 2 AgroBioTech Research Centre, Slovak University of Agriculture in Nitra, Tr. A. Hlinku 2, 949 76 Nitra, Slovakia; michal.duracka@uniag.sk; 3 Institute of Horticulture, Faculty of Horticulture and Landscape Engineering, Slovak University of Agriculture in Nitra, Tr. A. Hlinku 2, 949 76 Nitra, Slovakia; miroslava.kacaniova@uniag.sk; 4 School of Medical and Health Sciences, University of Economics and Human Sciences in Warsaw, Okopowa 59, 010 43 Warsaw, Poland

**Keywords:** methylxanthines, cryopreservation, bull, semen, cryocapacitation, bacteria, protein kinases

## Abstract

Theobromine (TBR) is a methylxanthine known for its bronchodilatory and stimulatory effects. This research evaluated the vitality, capacitation patterns, oxidative characteristics, microbial profile and expression of capacitation-associated proteins (CatSper1/2, sodium bicarbonate cotransporter [NBC], protein kinases A [PKA] and C [PKC] and adenylate cyclase 10 [ADCY10]) in cryopreserved bovine spermatozoa (n = 30) in the absence (cryopreserved control [Ctrl_C_]) or presence of different TBR concentrations (12.5, 25, and 50 µM) in egg yolk extender. Fresh ejaculate served as a negative control (Ctrl_N_). Significant post-thaw maintenance of the sperm motility, membrane and DNA integrity and mitochondrial activity (*p* < 0.001) were recorded following the administration of 25 μM and 50 μM TBR, then compared to Ctrl_C_. All groups supplemented with TBR exhibited a significantly lower percentage of prematurely capacitated spermatozoa (*p* < 0.001) than Ctrl_C_. Significantly decreased levels of global reactive oxygen species (ROS), hydrogen peroxide and hydroxyl radicals were observed in the presence of 25 μM and 50 μM TBR (*p* < 0.01). Western blot analysis revealed that supplementation with 50 μM TBR significantly prevented the loss of NBC and ADCY10 (*p* < 0.01), while all TBR doses stabilized the levels of PKC (*p* < 0.05 at 50 μM TBR; *p* < 0.001 at 12.5 μM and 25 μM TBR). In summary, we suggest that TBR is effective in protecting the spermatozoa during the cryopreservation process through its potential to stimulate energy synthesis while preventing ROS overproduction and the loss of proteins involved in the sperm activation process.

## 1. Introduction

Artificial insemination (AI) represents one of the major milestones in the evolution of livestock production because it has, together with cryopreservation and the long-term storage of spermatozoa, notably improved the worldwide distribution of genetically superior animals, thus helping with food production in a world with an ever-increasing population [1,2]. Nowadays, semen can be preserved via different cryopreservation procedures, such as slow or rapid programmable freezing and vitrification. The most common process relies on slow freezing, which is characterized by gradual cooling and temperature reduction by 1–2 °C/min to −196 °C. The success of cryopreservation depends on numerous factors, including the type of cryoprotectant, the extent of cell dehydration, equilibration time and the cooling, packaging and thawing rates [3,4].

Despite the numerous benefits of cryopreservation, a significant number of spermatozoa lose their fertilizing potential due to cryoinjury and the presence of freezing stressors that render them unsuitable for AI. Several factors are responsible for a decreased quality of frozen–thawed spermatozoa, such as ice crystal formation, generation of reactive oxygen species (ROS), energetic deficiency, osmotic imbalance and protein denaturation. Following thawing, spermatozoa may exhibit structural and biochemical changes resembling capacitation, a process known as cryocapacitation or cryo-induced capacitation [5].

The fertilization journey of spermatozoa starts shortly after their entrance to the female reproductive tract, leading to the activation of a series of biochemical and membrane modifications that allow male gametes to undergo acrosomal exocytosis, penetrate the follicular structures and fuse with the ovum at the end. These modifications begin with the removal of cholesterol from the sperm membrane and the increase of cyclic adenosine monophosphate (cAMP), intracellular pH and bicarbonate (HCO_3_^−^) and calcium (Ca^2+^) ions. Later, cAMP will trigger protein kinase A (PKA), while elevated HCO_3_^−^ and Ca^2+^ concentrations will stimulate soluble adenylyl cyclase (sAC), leading to the phosphorylation of the target proteins responsible for sperm activation (also known as hyperactivation) [6,7]. Hyperactivation is defined as a late event of sperm capacitation and is associated with an increased beat frequency of the sperm flagellum, lateral displacement of the sperm head and increased sperm velocity [8].

It has been reported that capacitated buffalo spermatozoa present with the same tyrosine phosphorylation patterns as bovine spermatozoa, while the loss of sperm surface proteins during cryopreservation may be similar to changes consistent with physiological capacitation. As such, frozen–thawed spermatozoa are in a partially capacitated state as a result of modifications to their membrane caused by low temperatures [9,10,11].

During cryopreservation, ROS are more likely to be generated, resulting in oxidative insults that will impair the sperm functionality. Furthermore, the sperm antioxidant system cannot counterbalance high ROS amounts, resulting in the development of oxidative stress (OS) and subsequent poor post-thaw semen quality [12,13]. To be able to perform normal functions, such as capacitation, hyperactivation and acrosome reaction, spermatozoa need a certain level of ROS generated by mitochondria. Nevertheless, spermatozoa exposed to high ROS concentrations may exhibit alterations in their motility patterns and fertilization ability due to mitochondrial dysfunction, plasma membrane disintegration, loss of acrosome or DNA fragmentation [14,15]. The molecules most vulnerable to oxidative damage are lipids. In general, polyunsaturated fatty acids (PUFAs) provide fluidity to the sperm plasma membrane. PUFAs attacked by ROS will undergo lipid peroxidation (LPO), which causes the loss of membrane fluidity and the impairment of membrane-bound enzymes and ion channels, as well as the formation of alkyl and peroxyl lipid radicals [16,17].

The susceptibility of sperm to oxidation depends on a number of factors, including the membrane structure, the number of mitochondria and endogenous antioxidant capacity. Since spermatozoa lack a robust inherent antioxidant system, it is crucial to preserve their fertilization ability by improving the antioxidant capacity of the extender. More specifically, supplementation of natural antioxidants stemming from plant-based substances and extracts with a wide spectrum of beneficial properties has been shown to improve sperm quality and fertilization ability [18,19,20]. Biomolecules of natural origin are increasingly being found as effective antioxidant supplements due to their high bioactivity and bioavailability, even at low doses, as well as to their low cytotoxicity against synthetic antioxidants [1,5]. Several alternative biomolecules, which may significantly ameliorate or prevent cryo-induced structural or functional damage to spermatozoa, have been already studied, including curcumin [21], lycopene [22], epicatechin [23], kaempferol [24] and resveratrol [25]. In addition to their ability to scavenge ROS and modulate the intrinsic antioxidant molecules, natural bioactive compounds exhibit antibacterial activity as well as remarkable antiapoptotic and cytomodulating properties, according to previous in vitro reports [26,27,28].

Theobromine (TBR) or 3,7-dimethylxanthine is a major methylxanthine alkaloid present in cocoa beans, coffee, tea and chocolate. It is well known for its vasodilative, analgetic and diuretic properties. At the same time, it has been suggested that the purine base analogue of TBR modulates spermatogenesis and the biosynthesis of testosterone [29,30]. Furthermore, it was reported that methylxanthines are able to maintain intracellular levels of cAMP by acting as phosphodiesterase (PDE) inhibitors, sperm motility enhancing agents or capacitation effectors [31]. However, the possible cryoprotective properties of TBR still need further investigation.

This study strove to assess the impact of TBR on the functional, oxidative and protein markers of frozen–thawed bovine spermatozoa in the context of an improvement of the cryopreservation potential to ensure a high-quality post-thaw sample for artificial insemination. We specifically focused on conventional sperm quality parameters, including motility and membrane, acrosome and DNA integrity. These were coupled with advanced sperm quality tests, including mitochondrial membrane potential and capacitation patterns. The potential antioxidant properties of theobromine were evaluated with the help of spectrophotometric, luminometric and fluorescent assays designed to quantify the levels of global ROS as well as those of major ROS types. The plate dilution method and matrix-assisted laser desorption/ionization time-of-flight (MALDI–TOF) mass spectrometry were used to assess the potential of TBR to prevent the spread of bacteria in cryopreserved semen. Finally, we evaluated the ability of TBR to prevent cryo-induced capacitation by monitoring the protein levels of selected capacitation-associated markers, including cation channels of sperm (CatSper) 1/2, sodium bicarbonate cotransporter (NBC), protein kinase A (PKA), protein kinase C (PKC) and adenylyl cyclase 10 (ADCY10).

## 2. Materials and Methods

### 2.1. Sample Collection and Freezing Procedure

Ejaculates were obtained from 30 healthy and sexually mature bulls using an artificial vagina at a local breeding facility (Slovak Biological Services, a.s., Nitra, Slovakia). Before cryopreservation, each semen specimen was divided into five equal aliquots as follows: the negative (native) control was represented by fresh, untreated ejaculates diluted to 44 × 10^6^ spermatozoa/mL in phosphate saline buffer (PBS; without calcium and magnesium chloride; Sigma-Aldrich, St. Louis, MO, USA); the positive (cryopreserved) control was processed in the absence of TBR and the experimental groups were supplemented with different concentrations of TBR (12.5, 25 and 50 µM; Sigma-Aldrich, St. Louis, MO, USA) dissolved in DMSO (dimethyl sulfoxide; Sigma-Aldrich, St. Louis, MO, USA). The selected concentration range was based upon the outcomes from our previous standardization studies [32,33]. The final DMSO concentration was 0.1% in each cryopreserved group. Samples for cryopreservation were diluted to 44 × 10^6^ spermatozoa/mL with a cryoprotective medium that consisted of Triladyl (Minitub GmbH, Tiefenbach, Germany), 20% (*w*/*v*) fresh egg yolk, Tris(hydroxylmethyl)aminomethane (Sigma-Aldrich, St. Louis, MO, USA), citric acid, glycerol, buffers, distilled water and antibiotics (500 µg of streptomycin/mL; 500 IU penicillin/mL; Sigma-Aldrich, St. Louis, MO, USA). The diluted semen was loaded into 0.25 mL French straws (with a final concentration of 11 × 10^6^ spermatozoa/straw), cooled down to 4 °C/2 h, frozen using a digital freezing machine (Digitcool 5300 ZB 250; IMV, France) at −3 °C/min. from 4 to −10 °C; −40 °C/min. from −10 °C to −100 °C; −20 °C/min. from −100 °C to −140 °C and stored in liquid nitrogen at −196 °C for six weeks. For further analysis, frozen straws were thawed in a water bath at 37 °C for 2 min. The samples were then transferred into new sterile 1.5 mL tubes, washed and centrifuged three times at 300× *g* for 10 min with 500 µL PBS [23,34].

### 2.2. Qualitative Sperm Parameters

The percentage of motile spermatozoa (motility) was assessed using Computer-Assisted Sperm Analysis (Version 14.0 TOX IVOS II., Hamilton-Thorne Biosciences, Beverly, MA, USA) and the Animal Motility program (Hamilton-Thorne Biosciences, Beverly, MA, USA) [23,34].

Mitochondrial activity was evaluated with a cytofluorimetric assay using the fluorescent dye JC-1 (Cayman Chemical, Ann Arbor, MI, USA), which has the ability to pass through the inner mitochondrial membrane. The fluorescent signal was measured using the combined spectro-fluoro-luminometer Glomax Multi^+^ (Promega, Madison, WI, USA) [23,34].

Fluorometry was used to assess sperm membrane integrity. The cells were stained with CFDA (carboxylfluorescein diacetate; Sigma-Aldrich, St. Louis, MO, USA) to quantify cellular esterase activity, which is an indicator of cell viability. Once CFDA enters the cell, esterases will cleave its acetoxymethyl groups, resulting in the formation of highly fluorescent 5-carboxyfluorescein that remains trapped within the cells, indicating their viability as only an intact membrane can maintain the cytoplasmic environment necessary to support esterase activity. The staining protocol also included PI (propidium iodide; Sigma-Aldrich, St. Louis, MO, USA), which identifies dead cells, and the nucleic acid dye DAPI (4′6-diamidine-2-phenylindole; Sigma-Aldrich, St. Louis, MO, USA) to indicate the total number of spermatozoa. Quantification of membrane integrity was performed with the Glomax Multi^+^ (Promega, Madison, WI, USA) [23,34].

Acrosomal integrity was evaluated using a PNA-lectin (FITC conjugate, Sigma-Aldrich, St. Louis, MO, USA) staining procedure without a permeabilization step, leading to PNA binding to the surface of acrosome-reacted spermatozoa. Hence, PNA-positive cells were considered to be acrosome-reacted. Similar to the previous analysis, the combined spectro-fluoro-luminometer Glomax Multi^+^ (Promega, Madison, WI, USA) was used [23,34].

For the analysis of the sperm DNA fragmentation index, we used the SCD (chromatin-dispersion) assay via the Halomax^®^ commercial kit (Halotech, Madrid, Spain). The samples were processed following the manufacturer’s instructions and at least 300 spermatozoa were examined under a fluorescent microscope (Leica Microsystems, Wetzlar, Germany) using a ×40 objective [34].

### 2.3. Assessment of the Sperm Capacitation Patterns

The capacitation status of spermatozoa was examined using a chlortetracycline (CTC) assay [23,35] under a fluorescent microscope (Leica Microsystems, Wetzlar, Germany) with a ×40 objective. According to the CTC fluorescence pattern, spermatozoa were divided into three categories as follows: “F” pattern for non-capacitated, “B” pattern for capacitated and “AR” pattern for acrosome-reacted spermatozoa. A minimum of 200 spermatozoa per sample were evaluated.

### 2.4. Evaluation of the Oxidative Profile

Intracellular and extracellular global ROS generation was assessed using a luminol-dependent chemiluminescence assay (Sigma-Aldrich, St. Louis, MO, USA) as previously outlined [34,36].

The evaluation of superoxide production was performed using a colorimetric nitroblue-tetrazolium (NBT) test. The optical density measurement was performed at wavelengths of 620/570 nm with the help of a Glomax microplate photometer (Promega, Madison, WI, USA) [23].

For the quantification of hydrogen peroxide, we used the fluorescent Amplex^®^ Red reagent (Thermo Fisher Scientific; Walthman, MA, USA), while levels of the hydroxyl radical were evaluated by APF (aminophenyl fluorescein; Thermo Fisher Scientific; Walthman, MA, USA). Global ROS and hydrogen peroxide, as well as hydroxyl radical production, were measured using the combined spectro-fluoro-luminometer Glomax Multi^+^ (Promega, Madison, WI, USA) [23,34].

### 2.5. Western Blotting

Before the assay, all samples were treated with single-layer 70% Percoll^®^ Plus (Sigma-Aldrich, St. Louis, MO, USA) as previously published [37]. Then, RIPA lysis buffer (Sigma-Aldrich, St. Louis, MO, USA), mixed with a protease inhibitor (Sigma-Aldrich, St. Louis, MO, USA), was added to the samples for overnight lysis at 4 °C. Next, the samples were centrifuged (5000× *g*, 10 min) and the total protein concentration was quantified using a commercially available kit (DiaSys, Holzheim, Germany) and the RX Monza biochemical analyzer (Randox, Crumlin, UK) [23]. All samples used for the assay were adjusted to a uniform protein concentration using a Laemmli sample buffer (BioRad, Hercules, CA, USA).

For the polyacrylamide gel electrophoresis (PAGE), randomly selected samples were treated with β-mercaptoethanol and boiled (95 °C/10 min). Then, 20 μg of protein from each selected sample was loaded onto readily available Mini-PROTEAN TGX stain-free polyacrylamide gels (BioRad, Hercules, CA, USA) and the proteins were separated with SDS PAGE. Subsequently, the gels were visualized with the ChemiDoc Imaging System (BioRad, Hercules, CA, USA) for loading uniformity. The Trans-Blot Turbo Transfer System (BioRad, Hercules, CA, USA) was used for the transfer of the gels to PVDF membranes (Trans-Blot Turbo Transfer Packs; BioRad, Hercules, CA, USA). The resulting membranes were washed with tris-buffered saline (TBS) and blocked with 5% milk (Sigma-Aldrich, St. Louis, MO, USA) in TBS/0.1% Tween-20 (Sigma-Aldrich, St. Louis, MO, USA) for 2 h at laboratory temperature [36]. Subsequently, all membranes were incubated at 4 °C overnight with primary antibodies based on their complementarity with selected proteins, as listed in Table 1.

Following overnight incubation with the primary antibodies, all the membranes were washed in 1% milk TBS/0.2% Tween-20 for 5 × 10 min and exposed to the secondary antibody (ECL anti-rabbit IgG, horseradish peroxidase-linked species-specific whole antibody, #NA934; GE Healthcare, Chicago, IL, USA) for 1 h. Then the membranes were washed again in TBS/0.2% Tween-20 for 5 × 10 min and incubated with ECL substrate (GE Healthcare, Chicago, IL, USA) for 1 h, followed by visualization using the ChemiDoc Imaging System and analysis with Image Lab software (version 6.1; BioRad, Hercules, CA, USA) [36].

### 2.6. Bacteriological Analysis and Identification of Bacteria

Determination of the bacterial count in the control and experimental groups was performed using the plate dilution method and expressed through colony-forming units (CFU). All samples were diluted and inoculated onto tryptic soy agar and blood agar (Sigma-Aldrich, St. Louis, MO, USA) and incubated under aerobic conditions to obtain pure cultures for identification purposes, as previously published [38]. Individual bacterial colonies were processed and identified with a MALDI–TOF Biotyper mass spectrometer (Brucker Daltonics, Bremen, Germany) using the Microflex LT instrument and the flexControl software version 3.4. [39]. The MALDI Biotyper Bruker Taxonomy database (Bruker Daltonics, Bremen, Germany) was used to evaluate the collected spectra.

### 2.7. Statistics

The results obtained were assessed with the GraphPad Prism program (version 8.4.3; GraphPad Software, La Jolla, CA, USA). One-way ANOVA and Tukey’s multiple comparison test were applied. The level of significance was set at * *p* < 0.05; ** *p* < 0.01; *** *p* < 0.001 and **** *p* < 0.0001.

## 3. Results

### 3.1. Sperm Viability

Following thawing (Table 2), a significant decrease (*p* < 0.0001) in sperm motility was recorded in the nontreated cryopreserved control group against the native control (Ctrl_N_) and TBR-treated groups. Increasing concentrations of TBR led to a significant dose-dependent improvement (*p* < 0.001) of the post-thaw motility in all experimental groups in comparison to the cryopreserved control (Ctrl_C_).

With respect to membrane integrity (Table 2), a significant preservation of the membrane stability was observed in all groups treated with TBR (25 and 50 µM TBR, *p* < 0.001 and 12.5 µM TBR, *p* < 0.01) in comparison to Ctrl_C_.

A significantly higher (*p* < 0.0001) presence of necrotic spermatozoa (Table 2) was observed in Ctrl_C_ when compared to Ctrl_N_. TBR supplementation exhibited significant protective effects against cell necrotic disintegration (25 and 50 µM TBR, *p* < 0.001 and 12.5 µM TBR, *p* < 0.01) when compared to the untreated cryopreserved control.

As indicated in Table 2, a significant decline (*p* < 0.001) in acrosomal integrity was detected in Ctrl_C_ as opposed to Ctrl_N_. Similar to the previous parameters, TBR treatment had a significant positive impact (*p* < 0.01 in the cases of 25 and 50 µM TBR; *p* < 0.05 with respect to 12.5 µM TBR) on acrosome integrity in comparison to Ctrl_C_.

Furthermore, a significant decrease in post-thaw mitochondrial activity was recorded in Ctrl_C_ when compared to Ctrl_N_ (*p* < 0.0001). On the contrary, all TBR doses significantly (*p* < 0.001) preserved the mitochondrial activity in the experimental groups (Table 2) in comparison with the cryopreserved control.

Finally, the beneficial effects of TBR were also confirmed in the case of sperm DNA integrity (Table 2), where all TBR concentrations significantly reduced (*p* < 0.001) the level of DNA cryodamage in the experimental groups in comparison to Ctrl_C_.

### 3.2. Sperm Capacitation Patterns

A significantly increased percentage of capacitated spermatozoa (“B” pattern) was observed in Ctrl_C_ as opposed to the Ctrl_N_ (*p* < 0.0001) and TBR-administered (*p* < 0.001) groups (Table 3). A significant decrease (*p* < 0.05) in acrosome-reacted spermatozoa (“AR” pattern) was recorded in the experimental group enriched with 25 µM TBR in comparison with Ctrl_C_. The groups supplemented with 12.5 and 50 µM of TBR exhibited a slight decline in spermatozoa that had undergone an acrosome reaction, although without significant differences.

### 3.3. Oxidative Profile

The data obtained reveal a significant increase (*p* < 0.0001) in the global ROS levels in Ctrl_C_ in comparison to Ctrl_N_ (Table 4). On the other hand, all groups supplemented with TBR exhibited a significant decrease (*p* < 0.01) in ROS production against Ctrl_C_.

In the case of superoxide generation (Table 4), a significant increase in its levels was detected in the Ctrl_C_ group (*p* < 0.001) in comparison with Ctrl_N_. All experimental groups enriched with TBR presented with a slight decrease in superoxide, although without significant differences.

Similar phenomena were observed in the cases of the production of hydrogen peroxide and the hydroxyl radical (Table 4). Significantly higher (*p* < 0.0001) concentrations of both reactive intermediates were recorded in the Ctrl_C_ group when compared to Ctrl_N_. Supplementation with TBR significantly decreased (*p* < 0.01) the concentration of both free radicals in the experimental groups treated with 25 and 50 µM TBR against the untreated Ctrl_C_ group.

### 3.4. Western Blot

**Figure 1:**
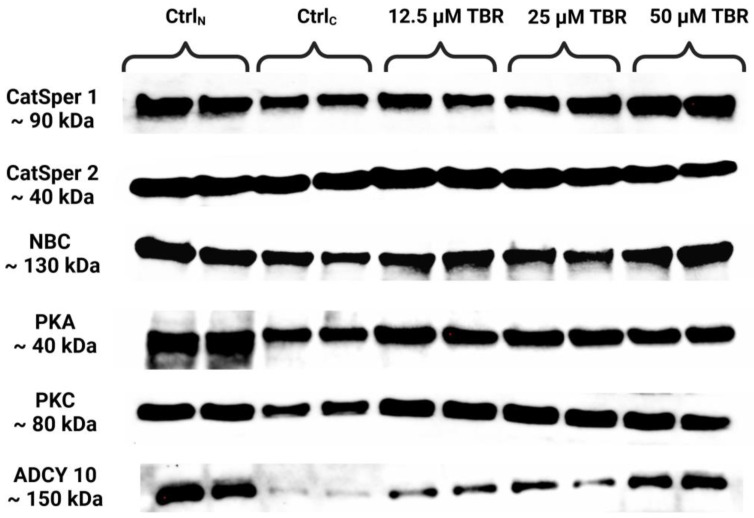
Protein levels of the cation channels of sperm isoforms 1 and 2 (CatSper1 and CatSper2), sodium bicarbonate cotransporter (NBC), protein kinase A (PKA), protein kinase C (PKC) and adenylyl cyclase 10 (ADCY10) in bovine spermatozoa in fresh state and cryopreserved in the absence or presence of selected theobromine (TBR) doses, assessed by Western blotting. Original photos of the gels and blots are available as Supplementary Materials. Created with BioRender.com (accessed on 27 August 2024).

Semiquantification of the levels of selected capacitation-associated proteins was performed using the Western blot technique (Figure 1).

**Figure 2:**
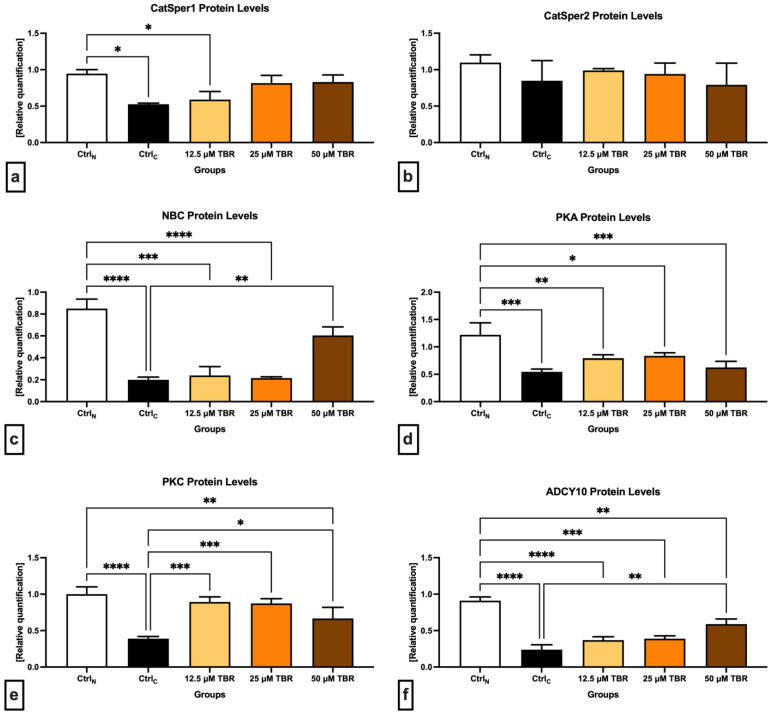
Graphical representation of the relative quantification of the CatSper1 (**a**), CatSper2 (**b**), NBC (**c**), PKA (**d**), PKC (**e**) and ADCY 10 (**f**) proteins in bovine spermatozoa (n = 30) in fresh state (native control (Ctrl_N_)) and cryopreserved in the absence (cryopreserved control (Ctrl_C_)) or presence of selected theobromine (TBR) doses. Mean ± S.D. * *p* < 0.05; ** *p* < 0.01; *** *p* < 0.001; **** *p* < 0.0001.

The data obtained revealed a significant decline (*p* < 0.05) in the CatSper1 protein quantity (Figure 2a) in the cryopreserved control, as well as in the experimental group supplemented with 12.5 µM TBR, when compared to Ctrl_N_. A nonsignificant improvement in the protein levels was observed following the administration of 25 and 50 µM of TBR in comparison to Ctrl_C_.

In comparison with Ctrl_N_, decreased protein levels in the cryopreserved control were also observed in the case of CatSper2, although they were not statistically significant (Figure 2b). Similar to the CatSper1 protein, a nonsignificant improvement in the CatSper2 protein levels was recorded in the experimental groups in comparison to Ctrl_C_, particularly when 12.5 and 25 µM TBR were administered.

In the case of the Na^+^/HCO_3_^−^ cotransporter (Figure 2c), a significant decline of its protein levels was observed in the Ctrl_C_ group (*p* < 0.0001) as well as in the groups supplemented with 12.5 µM TBR (*p* < 0.0001) and 25 µM of TBR (*p* < 0.001) when compared with Ctrl_N_. At the same time, the presence of 50 µM TBR was able to significantly stabilize (*p* < 0.01) the protein levels of the Na^+^/HCO_3_^-^ cotransporter in comparison to the Ctrl_C_ group.

In the meantime, all the cryopreserved groups, including the TBR-treated groups, presented with significantly lower PKA protein levels in comparison with Ctrl_N_ (*p* < 0.001 in the case of Ctrl_C_ and 50 µM TBR; *p* < 0.01 concerning 12.5 µM TBR; and *p* < 0.05 in relation to 25 µM TBR). None of the selected TBR doses was able to significantly affect the PKA protein levels in comparison with Ctrl_C_ (Figure 2d).

Similar to PKA, the lowest levels of the PKC protein were found in the cryopreserved control; they were significantly decreased in comparison to Ctrl_N_ (*p* < 0.0001) (Figure 2e). In the meantime, the administration of all TBR doses prevented the loss of the PKC protein in comparison to Ctrl_C_ (*p* < 0.001 in the case of 12.5 µM TBR and 25 µM TBR; and *p* < 0.05 concerning 50 µM TBR).

Finally, significantly decreased ADCY10 protein levels were recorded in all cryopreserved groups against the native control (*p* < 0.0001 with regards to Ctrl_C_ and 12.5 µM TBR; *p* < 0.001 in the case of 25 µM TBR; and *p* < 0.05 with respect to 50 µM TBR). Furthermore, the administration of 50 µM TBR led to a significant prevention of the loss of ADCY10 as opposed to the untreated cryopreserved control (*p* < 0.01).

### 3.5. Microbial Analysis

Bacterial identification (Table 5) revealed that TBR treatment may possibly suppress the presence of certain bacteria in the cryopreserved specimens following thawing. A significant decrease in the bacterial load was observed in all experimental groups, particularly in the case of 50 µM TBR, in comparison to the untreated Ctrl_C_ (*p* < 0.001) or the native Ctrl_N_ (*p* < 0.0001). Moreover, a significant decline (*p* < 0.0001) in the bacterial load was recorded in the Ctrl_C_ group when compared to Ctrl_N_.

## 4. Discussion

Even though sperm cryopreservation is a well-managed and technologically advanced process, particularly in the cattle industry, there are still considerable hurdles to be overcome to fully utilize its potential in breeding practice [1]. Given that spermatozoa present with lower intracellular water content and membranes that adapt their shape and movement to different conditions more readily, they should hypothetically be more resilient to low temperatures than somatic cells. Nonetheless, a substantial proportion of spermatozoa may be losing their integrity during the freeze–thaw procedure, rendering the semen sample unacceptable for artificial insemination [1,40,41].

The primary sperm structure that will respond to temperature fluctuations is the plasma membrane, which will become weaker, more fragile and rigid. The phospholipid layer will be detached and transmembrane proteins degraded, which may lead to the loss of the membrane’s integrity and semipermeability. Furthermore, cryopreservation has been shown to affect mitochondrial membrane fluidity, which may result in the loss of ATP and the leakage of ROS from dysfunctional mitochondria into the intracellular space. The axonemal proteins, critical for sperm motility and the mitochondrial enzymes associated with ATP synthesis, may undergo oxidation, which may result in energetic depletion and the loss of sperm motion activity [42,43,44,45].

Cryo-induced alterations to critical sperm structures may trigger changes mirroring natural capacitation while irreversibly compromising the cells, which will become more vulnerable upon their transfer to the female genital system and with a substantially reduced ability to accomplish fertilization. The data collected in this study indicate that exposure of bovine spermatozoa to cryogenic temperatures compromises all studied transmembrane channels responsible for the transport of HCO_3_^−^ and Ca^2+^, which should subsequently accelerate cAMP synthesis and activate PKA and tyrosine phosphorylases. This agrees with our earlier report [35] and with available studies on human [46] and rodent [47] spermatozoa. The underlying mechanisms of this phenomenon are multifactorial and are still subject to discussion. According to Flores et al. [48], such changes may be associated with aberrant interactions among mRNA and proteins, as well as with a more pronounced sensitivity of mRNA to cryodegradation. In the meantime, it has been suggested that transmembrane proteins could leak from already compromised membranes into the extracellular space [49]. Moreover, the loss of transmembrane proteins could be associated with an increased presence of already damaged or dead cells incapable of synthesizing more protein [41].

Furthermore, low PKA levels in the cryopreserved control may lie behind the loss of sperm motion and membrane fluidity, corroborating earlier studies on frozen–thawed bull [35] and fish [50] spermatozoa. PKA’s decline may result not only from alterations to the downstream molecular signaling cascade initiated by the CatSper and NBC activation that triggers it but also from damage to A-kinase anchoring proteins, which serve to bind PKA to its target proteins [51]. Low PKA levels could also be associated with damage to the acrosome in the cryopreserved control, as it has been previously suggested that any disruption to PKA anchoring proteins may lead to alterations in PKA localization within spermatozoa, resulting in premature acrosome reaction [52].

Several studies have emerged over the past years that emphasize the potential of biomolecules from natural sources to improve the quality of cryopreserved semen in a more efficient manner than traditional antioxidants, including ascorbic acid and tocopherol [16,53,54]. Methylxanthines are unique substances formed during the methylation of xanthine and include caffeine, theophylline (1,3-dimethylxanthine) and its isomer theobromine (3,7-dimethylxanthine), which is the major bitter alkaloid found in cocoa and chocolate. Based on previous findings, methylxanthines are able to increase the levels of intracellular cAMP and improve sperm motility by inhibiting phosphodiesterase enzymatic activity, which breaks phosphodiester bonds. In general, xanthines bind into the cAMP-binding catalytic pocket of phosphodiesterases by forming stable complexes. An increase in cAMP intracellular levels associated with higher ADCY10 activity, as observed in this study, may be responsible for higher sperm mitochondrial activity and oxygen uptake, which provide more energy for sperm movement [55,56,57,58].

It was hypothesized that methylxanthines could modulate the signaling pathway initiated by adenosine A_1_/A_2_ receptors, which are involved in sperm motion behavior, cAMP production, and protein phosphorylation, and may present with capacitative effects on spermatozoa. The lack of expression of adenosine receptors is related to delayed capacitation. Furthermore, methylxanthines have been reported to inhibit the activity of alkaline phosphatase in boar spermatozoa, which is related to the dephosphorylation of adenosine monophosphate [59,60]. The advantage of methylxanthines lies in their ability to combine with other biomolecules, such as polyphenols, without changing their original properties, thus accelerating their antioxidant effects. Werner et al. [61] designed a methylxanthine–polyphenol-based supplement for the protection of spermatozoa during the cryopreservation procedure. A combination of selected compounds increased post-thaw viability and decreased oxidative damage by modulating the levels of ROS and nitric oxide in human spermatozoa.

The antioxidant properties of theophylline (TP) and theobromine (TB) were confirmed based on their interactions with superoxide dismutase (SOD) in an earlier study by Wu et al. [62]. Both molecules form spontaneous complexes with SOD without changes in their antioxidant activity. Furthermore, the synergic properties of the TP-SOD and TB-SOD complexes exhibited inhibitory effects on the production of malondialdehyde (MDA) as the main product of LPO. This agrees with our results, where 25 and 50 µM TBR, in particular, improved the oxidative profile of cryopreserved spermatozoa.

Similar to our experimental outcomes, Bishist et al. [30] reported that the supplementation of selected methylxanthines, including pentoxiphylline, TP and TB, led to a higher preservation of the motility, membrane integrity and acrosome integrity of frozen–thawed buffalo spermatozoa. According to Okada et al. [63], dimethylxanthine treatment increased the sperm viability and the number of spermatozoa with intact acrosomes and membranes, as well as reducing LPO and leading to a lower incidence of oxidative insults to the DNA molecule. Moreover, Calza et al. [64] reported that methylxanthines, such as theophylline, recovered the post-thaw motility of surgically retrieved human spermatozoa. This agrees with a previous study by Ebner et al. [65], where theophylline supplementation allowed faster and more accurate selection of viable human testicular spermatozoa following thawing.

According to Belham et al. [66], short-term administration of theophylline (75 mg/kg for 14 days) to rats increased sperm motility but had no effect on sperm density or abnormal/dead sperm count in comparison with the nontreated control. On the other hand, long-term treatment of rats from 14 to 75 weeks of age with methylxanthines, such as theophylline, led to the development of severe or moderate testicular atrophy [67]. It was also reported that prolonged administration of high theophylline doses (800 ppm/day) may significantly increase the abnormal sperm count of rats, whereas shorter treatment periods had no adverse effects on sperm quality. These findings confirm that the use of methylxanthines is dose- and time-dependent and the specifics of its administration need to be carefully considered from case to case [68].

Currently, there is still a lack of knowledge on the mechanisms of action of methylxanthines, especially theophylline or theobromine, on the male gamete. Our experimental design, specifically TBR concentrations selected for in vitro treatment, was based on our previous experience with kaempferol [36,69], the chosen doses of which were able to preserve the levels of capacitation-associated proteins and improve the post-thaw quality parameters of cryopreserved bovine spermatozoa.

At the same time, the possible promising antimicrobial effects of methylxanthines were suggested in earlier reports. Mutual interactions between methylxanthines and antibiotics such as gentamycin increased their antimicrobial activity against *Staphylococcus aureus* and *Pseudomonas aureginosa* by reducing the minimal inhibitory concentration (MIC) [70,71,72], suggesting that theobromine could aid in preventing the bacterial contamination of cryopreserved semen samples with the advantage of a lower spermatotoxicity than that of conventional antibiotics.

We may assume that cryopreservation directly affects the functionality of the transmembrane channels and the sperm intracellular ionic environment, which may result in the reduced activity of sAC and PKA accompanied by a decline in cAMP concentration. Moreover, cryogenic temperatures may promote the transition of liquid membrane components into a gel-like state, which would modify membrane fluidity and integrity. All such phenomena may be responsible for a significant loss from the membrane of proteins, such as PKA and PKC, as well as phospholipids; this is then mirrored by an increased response of cells to the CTC assay and a higher proportion of “false-capacitated” (CTC “B” pattern) and acrosome-reacted spermatozoa (CTC “AR” pattern).

Based on our current findings, theobromine supplementation may lead to an improved post-thaw quality of spermatozoa at different levels. First, it acts as a phosphodiesterase inhibitor, which prevents the loss of cAMP as a source of energy for sperm metabolism. Second, the molecule may possibly inhibit the adenosine receptors to prevent premature cryo-induced capacitation, although its effects depend on an appropriate dosage. Moreover, the inhibition of alkaline phosphatase may prevent the dephosphorylation of cAMP, which in theory may restore the energy for ATP synthesis following thawing.

While the outcomes of this study seem promising, our experiments are limited to a rather homogeneous cohort of animals. We did select a milk breed for our experiments, which generally presents with a higher semen quality in comparison to beef breeds [73]. Therefore, we must agree that the robustness of our findings could have been increased by including samples from a wider variety of bulls across different breeds and geographical locations to ensure that the results were not specific to a particular population. At the same time, the recreation of our experimental design in animals that may present with a substandard semen quality even prior to the cryopreservation procedure could lead to more pronounced effects of theobromine on post-thaw sperm quality. However, such hypotheses are subject to future investigation.

## 5. Conclusions

In summary, our results suggest that a range of 25–50 µM TBR may improve the post-thaw viability of cryopreserved bovine spermatozoa and prevent the onset of cryo-induced capacitation. The antioxidant abilities of theobromine may reduce the risk of oxidative stress development by decreasing the global generation of ROS while stabilizing the membrane’s structural integrity. What is more, meticulously selected TBR doses may preserve the levels of capacitation-associated proteins in spermatozoa and exhibit promising antibacterial properties. Nevertheless, the actual fertilization ability of post-thaw bovine spermatozoa treated with TBR needs further investigation through either sperm penetration tests or insemination studies.

## Figures and Tables

**Table 1:** Primary antibodies used for the Western blot procedure.

Protein of Interest	Name	Cat. No.	Source	Dilution	Manufacturer
CatSper 1	CATSPER1 polyclonal antibody	#PA5-75788	rabbit	1:1000, 5% milk/TBS/0.1% Tween-20	Invitrogen, Waltham, MA, USA
CatSper 2	CATSPER2 polyclonal antibody	#PA5-41072	rabbit	1:1000, 5% milk/TBS/0.1% Tween-20	Invitrogen, Waltham, MA, USA
NBC	Anti-Na^+^/HCO_3_^−^ cotransporter polyclonal antibody	#AB3212-I	rabbit	1:500, 5% milk/TBS/0.1% Tween-20	EMD Millipore Corporation, Temecula, CA, USA
PKA	PKA α antibody	#PA5-17626	rabbit	1:1000, 5% milk/TBS/0.1% Tween-20	Invitrogen, Waltham, MA, USA
PKC	PKC alpha monoclonal antibody	#PA5-43049	rabbit	1:1000, 5% milk/TBS/0.1% Tween-20	Invitrogen, Waltham, MA, USA
ADCY10	ADCY10 polyclonal antibody	#PA5-43049	rabbit	1:1000, 5% milk/TBS/0.1% Tween-20	Invitrogen, Waltham, MA, USA

CatSper 1—cation channels of sperm isoform 1, CatSper 2—cation channels of sperm isoform 2, NBC—sodium bicarbonate cotransporter, PKA—protein kinase A, PKC—protein kinase C, ADCY—adenylyl cyclase 10.

**Table 2:** Vitality of bovine spermatozoa (n = 30) in fresh state (native control; Ctrl_N_) and cryopreserved in the absence (cryopreserved control (Ctrl_C_)) or presence of selected theobromine (TBR) concentrations.

Group/Parameter	Ctrl_N_	Ctrl_C_	12.5 µM TBR	25 µM TBR	50 µM TBR
Sperm motility [%]	93.70 ± 3.52	57.00 ± 2.86 ****^N^	70.30 ± 3.42 ***^N;^ ***^C^	77.20 ± 4.66 ***^N;^ ***^C^	77.90 ± 2.94 ***^N;^ ***^C^
Membrane integrity [%]	94.56 ± 1.67	67.77 ± 3.85 ****^N^	78.87 ± 3.96 ****^N;^ **^C^	81.14 ± 5.12 ****^N;^ ***^C^	80.23 ± 5.31 ****^N;^ ***^C^
Necrotic sperm [%]	3.06 ± 0.26	14.75 ± 2.45 ****^N^	8.44 ± 0.77 ***^N;^ **^C^	6.37 ± 0.42 **^N;^ ***^C^	6.99 ± 0.49 **^N;^ ***^C^
Acrosome integrity [%]	91.83 ± 3.47	69.76 ± 2.52 ***^N^	79.01 ± 7.10 **^N;^ *^C^	81.83 ± 5.37 *^N;^ **^C^	80.04 ± 5.27 *^N;^ **^C^
Mitochondrial activity [%]	3.16 ± 0.29	1.59 ± 0.16 ****^N^	2.61 ± 0.32 *^N;^ ***^C^	2.64 ± 0.26 *^N;^ ***^C^	2.63 ± 0.37 *^N;^ ***^C^
DNA damage [%]	6.13 ± 2.47	33.23 ± 2.58 ****^N^	18.40 ± 1.69 ***^N;^ ***^C^	16.14 ± 1.53 ***^N;^ ***^C^	17.09 ± 1.52 ***^N;^ ***^C^

* *p* < 0.05; ** *p* < 0.01; *** *p* < 0.001; **** *p* < 0.0001; ^N^—in comparison with native control (Ctrl_N_); ^C^—in comparison with cryopreserved control (Ctrl_C_).

**Table 3:** Capacitation patterns of bovine spermatozoa (n = 30) in fresh state (native control; Ctrl_N_) and cryopreserved in the absence (cryopreserved control (Ctrl_C_)) or presence of selected theobromine (TBR) concentrations.

Group/Parameter	Ctrl_N_	Ctrl_C_	12.5 µM TBR	25 µM TBR	50 µM TBR
Non-capacitated sperm [%]	87.86 ± 1.81	52.87 ± 3.32 ****^N^	71.01 ± 4.08 **^N;^ ***^C^	77.51 ± 3.10 **^N;^ ****^C^	76.26 ± 4.00 **^N;^ **^C^
Capacitated sperm [%]	6.12 ± 1.28	35.52 ± 4.66 ****^N^	17.34 ± 4.08 ***^N;^ ***^C^	14.17 ± 3.68 ***^N;^ ***^C^	14.58 ± 3.06 ***^N;^ ***^C^
Acrosome-reacted sperm [%]	6.02 ± 0.58	14.61 ± 1.44 **^N^	11.65 ± 1.03	8.32 ± 1.86 *^C^	9.17 ± 1.64

* *p* < 0.05; ** *p* < 0.01; *** *p* < 0.001; **** *p* < 0.0001; ^N^—in comparison with native control (Ctrl_N_); ^C^—in comparison with cryopreserved control (Ctrl_C_).

**Table 4:** Oxidative profile of bovine spermatozoa (n = 30) in fresh state (native control; Ctrl_N_) and cryopreserved in the absence (cryopreserved control (Ctrl_C_)) or presence of selected theobromine (TBR) concentrations.

Group/Parameter	Ctrl_N_	Ctrl_C_	12.5 µM TBR	25 µM TBR	50 µM TBR
ROS levels [RLU/s/10^6^ sperm]	4.93 ± 0.45	15.11 ± 0.49 ****^N^	10.94 ± 0.90 ***^N;^ **^C^	9.52 ± 0.49 ***^N;^ **^C^	9.39 ± 0.53 ***^N;^ **^C^
O_2_^−•^ levels [%]	100.00 ± 9.33	185.40 ± 23.85 ***^N^	128.80 ± 15.24	127.10 ± 10.48	127.80 ± 7.79
H_2_O_2_ levels [RFU/10^6^ sperm]	2.00 ± 0.68	7.62 ± 0.88 ****^N^	5.33 ± 0.92 **^N;^ *^C^	4.73 ± 0.35 *^N;^ **^C^	4.82 ± 0.87 *^N;^ **^C^
•OH levels [RFU/10^6^ sperm]	3.86 ± 0.61	15.85 ± 0.86 ****^N^	9.51 ± 0.81 **^N;^ **^C^	9.36 ± 1.03 **^N;^ **^C^	9.23 ± 0.87 **^N;^ **^C^

* *p* < 0.05; ** *p* < 0.01; *** *p* < 0.001; **** *p* < 0.0001; ^N^—in comparison with native control (Ctrl_N_); ^C^—in comparison with cryopreserved control (Ctrl_C_). O_2_^−•^—superoxide, H_2_O_2_—hydrogen peroxide, •OH—hydroxyl radical.

**Table 5:** The effects of theobromine (TBR) on the bacteriological profile of cryopreserved bovine spermatozoa.

Groups	Identified Bacteria (Sample Positivity)	Bacterial Count TSA (log_10_ CFU/mL)	Bacterial Count BA (log_10_ CFU/mL)
Ctrl_N_	*Arthrobacter koreensis* (5/30), *Kocuria rhizophila* (4/30), *Micrococcus luteus* (4/30), *Neisseria elongata* (3/30), *Rothia aeria* (4/30), *Rhodotorula mucilaginosa* (5/30), *Staphylococcus aureus* (2/30), *Staphylococcus epidermidis* (8/30), *Staphylococcus hominis* (12/30), *Staphylococcus warneri* (4/30)	9.22 ± 1.03	6.44 ± 0.51
Ctrl_C_	*Arthrobacter koreensis* (3/30), *Kocuria rhizophila* (2/30), *Micrococcus luteus* (2/30), *Staphylococcus aureus* (2/30), *Staphylococcus epidermidis* (3/30), *Staphylococcus hominis* (5/30), *Staphylococcus warneri* (2/30)	2.15 ± 0.49 ****^N^	2.10 ± 0.44 ****^N^
12.5 µM TBR	*Arthrobacter koreensis* (2/30), *Kocuria rhizophila* (2/30), *Micrococcus luteus* (1/30), *Staphylococcus aureus* (1/30), *Staphylococcus epidermidis* (3/30), *Staphylococcus hominis* (4/30), *Staphylococcus warneri* (2/30)	1.65 ± 0.13 ****^N;^ *^C^	1.54 ± 0.12 ****^N^
25 µM TBR	*Arthrobacter koreensis* (2/30), *Kocuria rhizophila* (1/30), *Micrococcus luteus* (1/30), *Staphylococcus aureus* (1/30), *Staphylococcus epidermidis* (2/30), *Staphylococcus hominis* (4/30), *Staphylococcus warneri* (1/30)	1.64 ± 0.10 ****^N;^ *^C^	1.51 ± 0.12 ****^N^
50 µM TBR	*Micrococcus luteus* (1/30), *Staphylococcus hominis* (2/30), *Staphylococcus warneri* (1/30)	1.48 ± 0.07 ****^N;^ ***^C^	1.37 ± 0.08 ****^N;^ *^C^

* *p* < 0.05; *** *p* < 0.001; **** *p* < 0.0001; ^N^—in comparison with native control (Ctrl_N_); ^C^—in comparison with cryopreserved control (Ctrl_C_). TSA—tryptic soy agar, BA—blood agar.

## Data Availability

The data presented in this study are available upon reasonable request from the corresponding author.

## Supplementary Materials

The following supporting information can be downloaded at: https://www.mdpi.com/article/10.3390/cells13201710/s1, Figure S1: representative photograph of the loaded gel; Figure S2: Original CatSper1 blot; Figure S3: Original CatSper2 blot; Figure S4: Original NBC blot; Figure S5: Original PKA blot; Figure S6: Original PKC blot; Figure S7: Original ADCY10 blot.
