# Supplementary figures and images for: Cryoprotective Potential of Theobromine in the Improvement of the Post-Thaw Quality of Bovine Spermatozoa
Source: Cells. 2024 Oct 16;13(20):1710. doi: 10.3390/cells13201710 (PMC11505711; doi:10.3390/cells13201710)

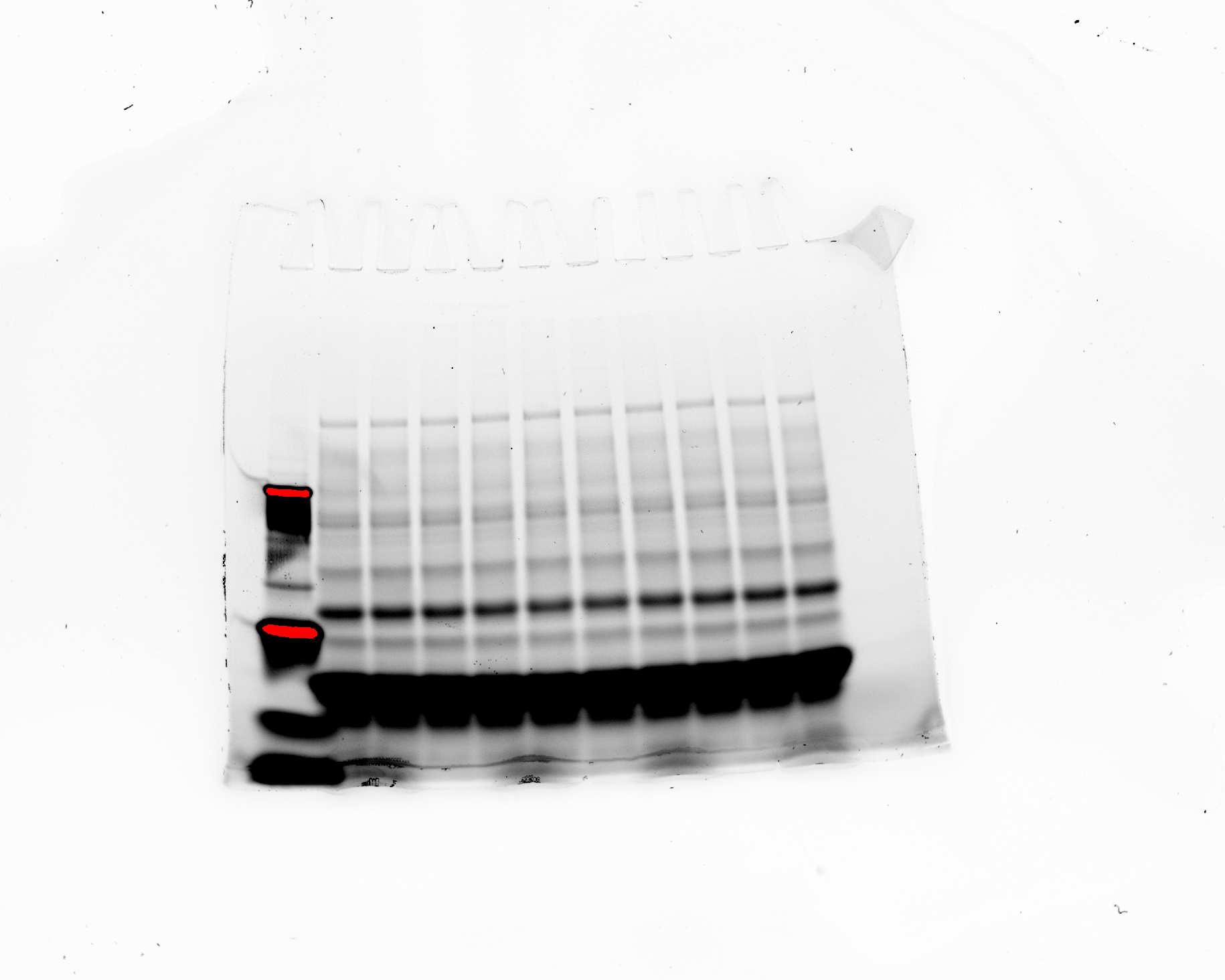

Supplement: Supplementary file 1 [file cells-13-01710-s001.zip › Figure S1.tif]

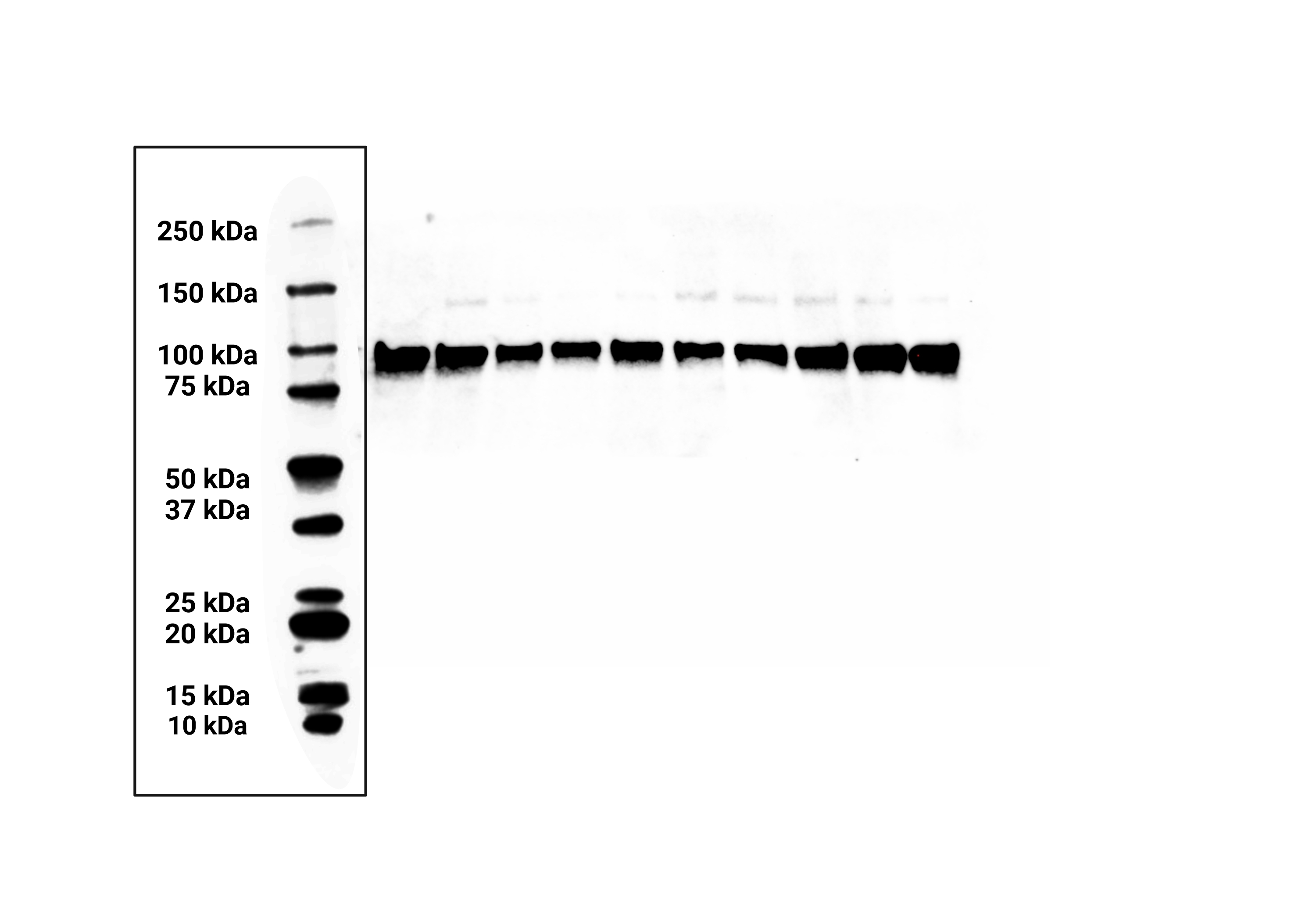

Supplement: Supplementary file 1 [file cells-13-01710-s001.zip › Figure S2.jpeg]

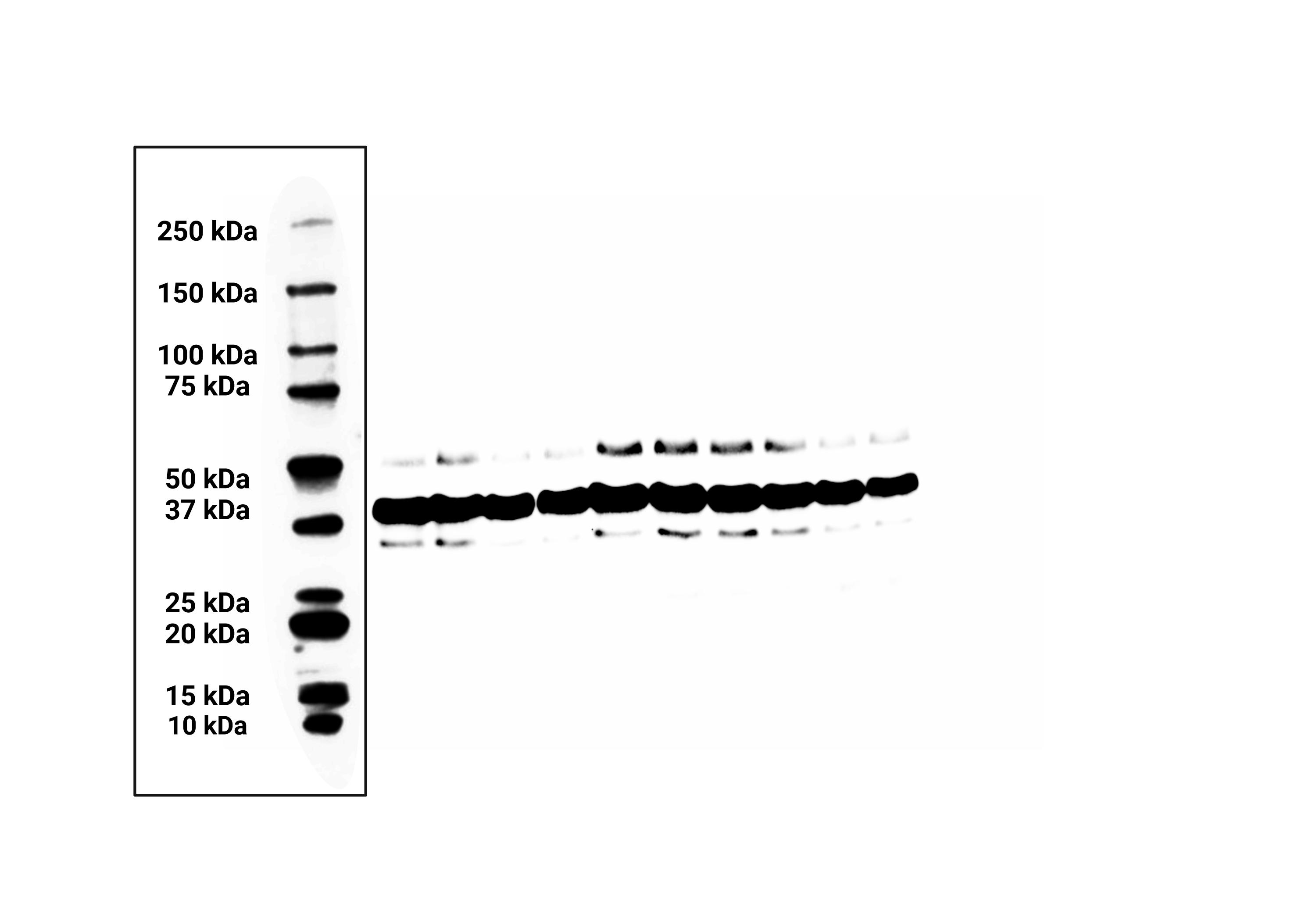

Supplement: Supplementary file 1 [file cells-13-01710-s001.zip › Figure S3.jpeg]

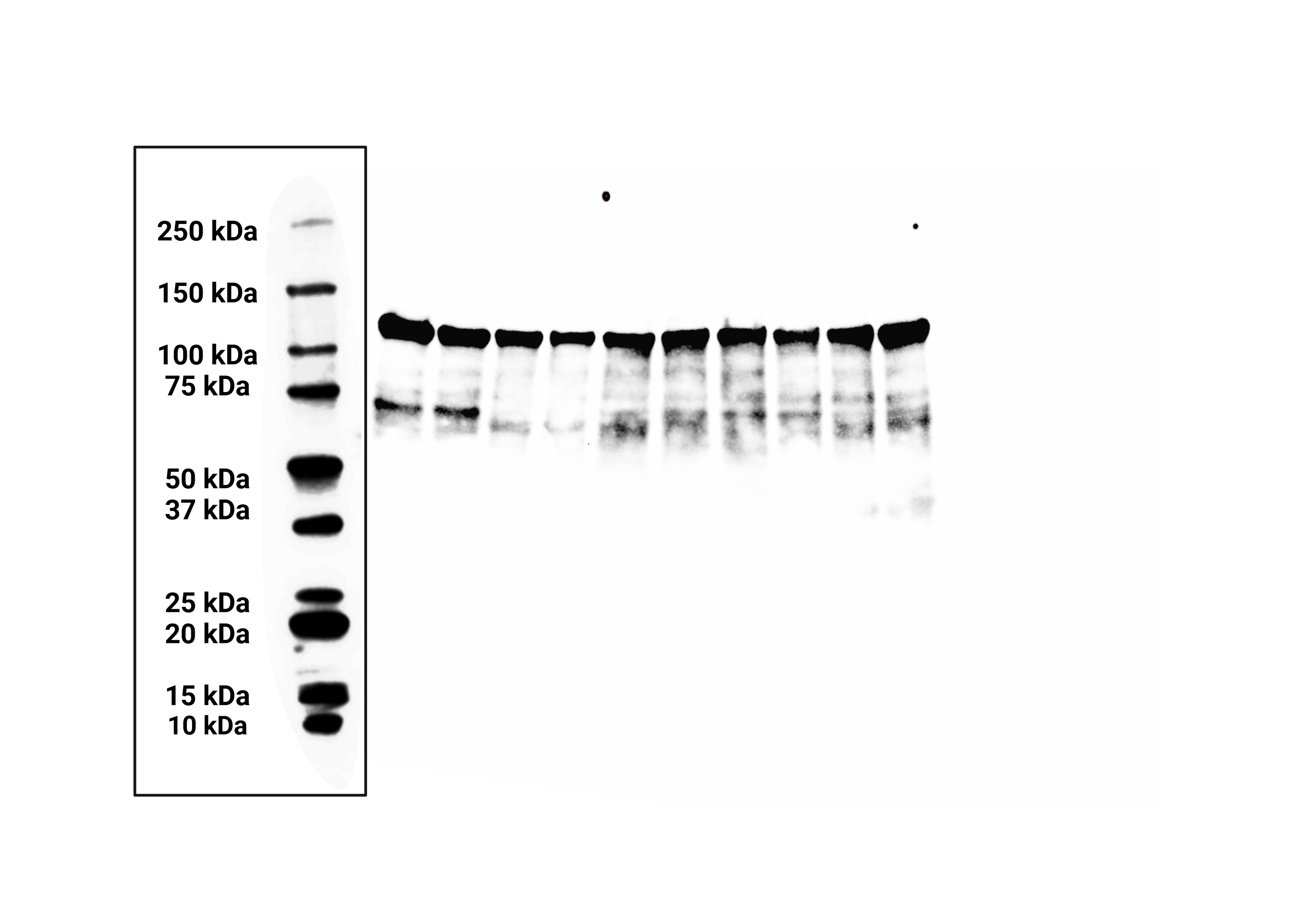

Supplement: Supplementary file 1 [file cells-13-01710-s001.zip › Figure S4.jpeg]

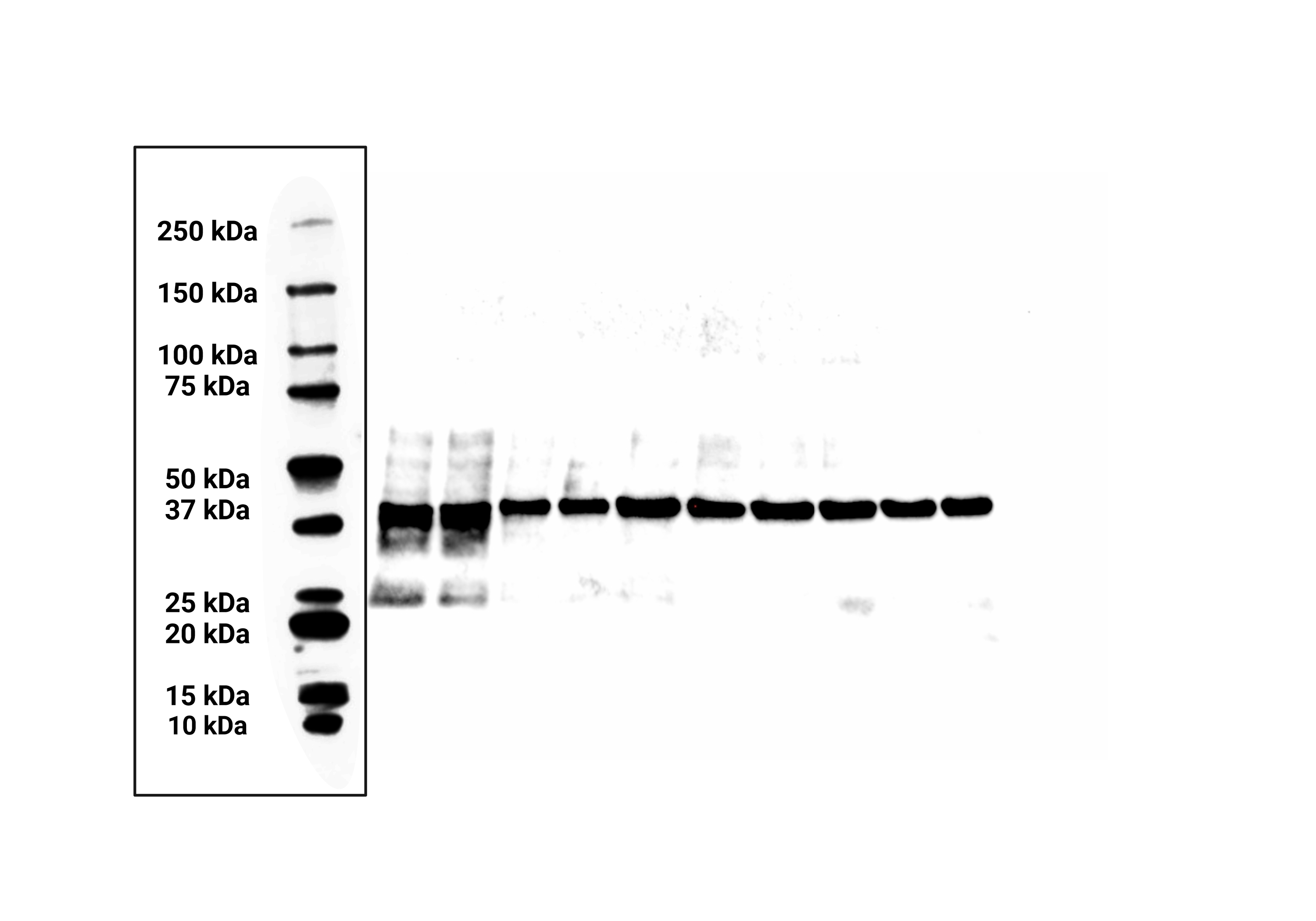

Supplement: Supplementary file 1 [file cells-13-01710-s001.zip › Figure S5.jpeg]

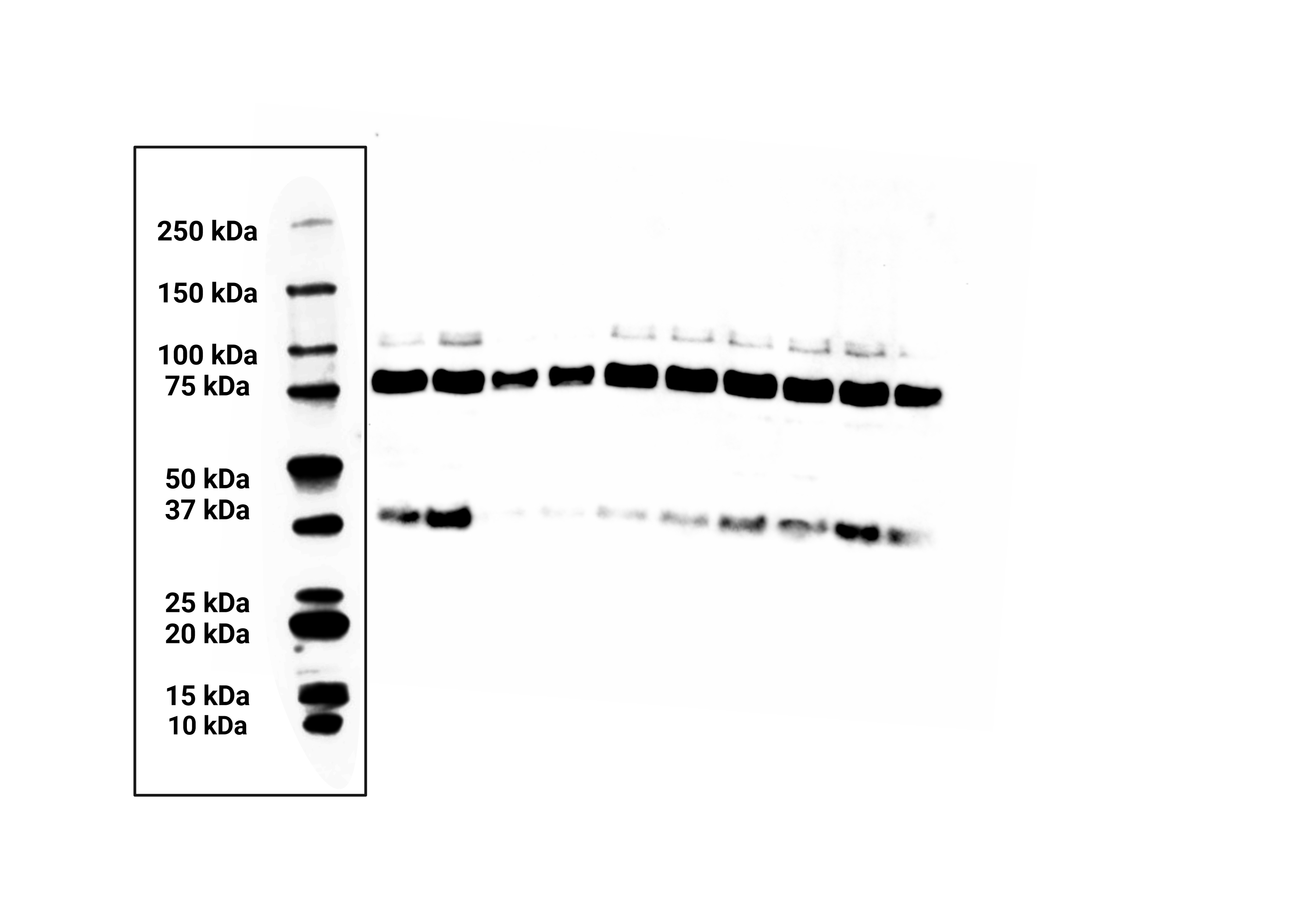

Supplement: Supplementary file 1 [file cells-13-01710-s001.zip › Figure S6.jpeg]

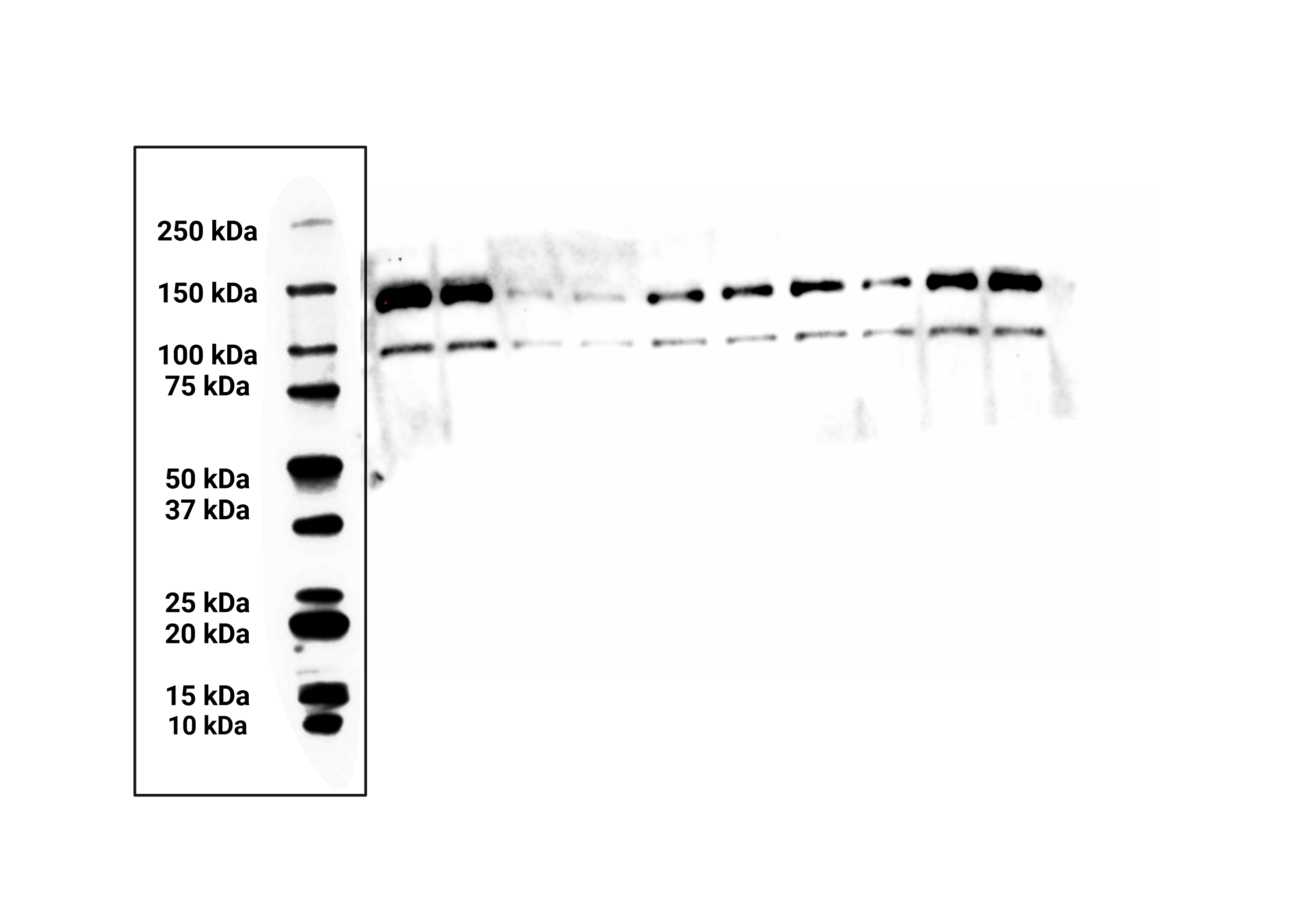

Supplement: Supplementary file 1 [file cells-13-01710-s001.zip › Figure S7.jpeg]
